# Characterization of Ethyl Acetate and Trichloromethane Extracts from *Phoebe* *zhennan* Wood Residues and Application on the Preparation of UV Shielding Films

**DOI:** 10.3390/molecules25051145

**Published:** 2020-03-04

**Authors:** Fangya Pan, Lin Chen, Lu He, Yongze Jiang, Jinqiu Qi, Hui Xiao, Yuzhu Chen, Xingyan Huang, Hongling Hu, Lihua Tu, Tiantian Lin, Gang Chen, Jianfeng Hao, Yinlong Xiao, Jiulong Xie

**Affiliations:** 1 College of Forestry, Sichuan Agricultural University, Chengdu 611130, China; panfangya@aliyun.com (F.P.); chenlin2018@aliyun.com (L.C.); HeLu53684@aliyun.com (L.H.); 72076@sicau.edu.cn (Y.J.); qijinqiu2005@aliyun.com (J.Q.); xh_b70405@126.com (H.X.); populus_zhu@163.com (Y.C.); hxy@sicau.edu.cn (X.H.); littlehhl@163.com (H.H.); iamtlh@163.com (L.T.); tlinsau@163.com (T.L.); g.chen@sicau.edu.cn (G.C.); haojf2005@aliyun.com (J.H.); 2 College of Environment, Sichuan Agricultural University, Chengdu 611130, China; xylsicau@163.com

**Keywords:** wood extract, polylactic acid, ethyl acetate, trichloromethane, UV shielding

## Abstract

In this work, ethyl acetate (EA) and trichloromethane (TR) extracts were extracted from *Phoebe* *zhennan* wood residues and the extracts were then applied to the preparation of UV shielding films (UV-SF). The results revealed that substances including olefins, phenols and alcohols were found in both EA and TR extracts, accounting for about 45% of all the detected substances. The two extracts had similar thermal stability and both had strong UV shielding ability. When the relative percentage of the extract is 1 wt% in solution, the extract solution almost blocked 100% of the UV-B (280–315 nm), and UV-A (315–400 nm). Two kinds of UV-SF were successfully prepared by adding the two extracts into polylactic acid (PLA) matrix. The UV-SF with the addition of 24 wt% of the extractive blocked 100% of the UV-B (280–315 nm) and more than 80% of the UV-A (315–400 nm). Moreover, the UV shielding performance of the UV-SF was still stable even after strong UV irradiation. Though the addition of extracts could somewhat decrease the thermal stability of the film, its effect on the end-use of the film was ignorable. EA extracts had less effect on the tensile properties of the films than TR extracts as the content of the extract reached 18%. The results of this study could provide fundamental information on the potential utilization of the extracts from *Phoebe zhennan* wood residues on the preparation of biobased UV shielding materials.

## 1. Introduction

Though extracts contain minor parts of the plant composition and are present in relatively small amounts compared to the main cell wall components, including cellulose, hemicellulose and lignin, they play vital roles in plant life and biochemical industries [1]. Plant extracts have been found to have many biological activities and utilizations. For example, extracts from oak had inhibition ability toward *Staphylococcus aureus* and *Candida albicans* [2]. The compounds isolated from *Nicandra john-tyleriana* could inhibit *Bacillus*, *Enterococcus*, *Escherichia*, *Listeria*, *Pseudomonas* and *Staphylococcus* strains [3]. Extracts from *Tossa jute* leaves, *Perilla* leaves, and *Fenugreek* seeds exhibited strong antioxidant activity [4,5,6,7,8]. Extracts were also reported to have biological activities such as anti-inflammatory activity [9,10,11,12], glycosidase inhibition activity [13], protective ability of the liver [14], and antiplatelet activity [15]. Because of the high values of plant extracts, techniques such as supercritical fluid have been explored in order to improve the extract yield and quality [16,17].

*Phoebe zhennan* is an evergreen large arbor belonging to the genus *Phoebe* (Lauraceae). It is mainly grown in the southwest Shanxi and Sichuan Province, northeast Yunnan, and northwest Hunan in China. As reported, the estimated volumes for *Phoebe zhennan* wood in Hunan, China in 2014 was 9.2 million m^3^ [18]. The special character of the *Phoebe zhennan* trees is their straight trunks and high-quality wood; therefore, it has been widely planted and used as urban landscape and rural garden trees because of its indispensable green and high growth rate. *Phoebe zhennan* wood resources from planted trees have become good resources for solid wood furniture manufacture due to the largely distributed artificial *Phoebe zhennan* plantations in southwest China, especially in Sichuan province. At present, research on *Phoebe zhennan* mainly focuses on wood identification [19], essential oil extraction and characterization [20], silviculture and genetic diversity [21]. With the rapid increase in the timber production from *Phoebe zhennan* plantations, solid wood furniture fabricated from *Phoebe zhennan* wood has risen in recent years in local regions in Sichuan, China, and large amounts of wood processing residues produced, which has been discarded as waste or combusted for energy resulting in resource waste or environmental issues. 

In order to address the resources waste issues from the artificial *Phoebe zhennan* industry, previous research studied the chemical compositions, antioxidant and UV/Vis absorptivity of essential oils from bark and leaf of *Phoebe zhennan* trees [22]. Comparative analysis in chemical compositions and physicochemical characterization of essential oils between the modern and ancient *Phoebe zhennan* wood was also conducted in previous research to provide detailed information for potential utilization of the essential oils [20]. However, due to the low yield of essential oils from the wood, the exploration of the solvent extracts from *Phoebe zhennan* wood seems to be a preferred approach to improve the value of the wood processing residues; to date, such research has not been reported. 

Since ultraviolet (UV) photons are very energetic in reaching the Earth’s surface and thus able to break chemical bonds and rearrange molecular structures [23], UV light can cause skin lesions [24], paper yellowing [25], plastic aging [26] and promote the oxidative degradation of foods [27]. Therefore, the development of UV shielding materials has attracted great interest; for example, a transparent TiO_2_ xerogel has been fabricated to protect wood from discoloration and degradation [28]. Carbon dots were used to enhance the UV-blocking properties of transparent nanocellulose films [29]. A simple one-pot method was used to prepare a UV protective film by adding alkali lignin and SiO_2_ into a polyurethane matrix [30]. Biobased UV protective films were also investigated. Organic lignin or modified lignin was mixed with polylactic acid (PLA) to fabricate the lignin-PLA UV protective films [31]. Valonea tannin with a purity of 68% was added to soya by-products to develop antioxidant and flexible packaging films, and the films also exhibited UV light absorption [32]. For comparison, the UV protective film prepared by using valine tannin and soya by-products had antioxidant activity; however, they had lower mechanical properties than that prepared from polylactic acid PLA upon addition of UV light absorbers. For PLA based UV protective films, PLA is mainly used as the plastic matrix because of its biodegradable character and its sustainable alternative to petrochemical-derived products. In order to expand the industrial applications of natural UV light absorber in thermoplastic industries, more explorations on the biobased PLA UV protective films should be made.

In our previous research, diethyl ether extracts were isolated from *Phoebe zhennan,* and UV protective films were successfully fabricated by adding the diethyl ether extracts into a polylactic acid matrix [33]. In later research, extracts from distilled water, methyl alcohol, ethanol, 3% HCl solution, 1% NaOH solution, acetone, diethyl ether, ethyl acetate, trichloromethane, a mixture of methylbenzene and ethanol (1:1) were also prepared, and their solution also showed UV light absorption ability. However, only extracts from diethyl ether, ethyl acetate and trichloromethane solvents can be well miscibility with the PLA matrix. The hypothesis may be that the successful preparation of UV protective films from extracts was largely dependent on the characteristics of the extracts from the various solvents. Therefore, in this study, the wood processing residues (sapwood of the wood xylem) from the *Phoebe zhennan* wood furniture industry were collected and subjected to ethyl acetate and trichloromethane solvent extraction. The extracts were condensed and thoroughly characterized by using gas chromatography-mass spectrometry (GC-MS), Fourier transform infrared spectroscopy (FTIR), thermogravimetric analysis (TGA), and UV-vis spectroscopy. The UV absorption performance of a diluted extract solution was also examined. The condensed extracts were applied to a PLA matrix in order to fabricate UV shielding film; thereafter, the UV absorption performance and UV stability of the fabricated UV shielding film were investigated. The aim of this study was to obtain fundamental information on the two extracts of *Phoebe zhennan* sapwood residues and to test the flexibility of the extracts on the preparation of biobased biodegradable UV protective materials.

## 2. Results and Discussion

### 2.1. Chemical Constituents

**Figure 1:**
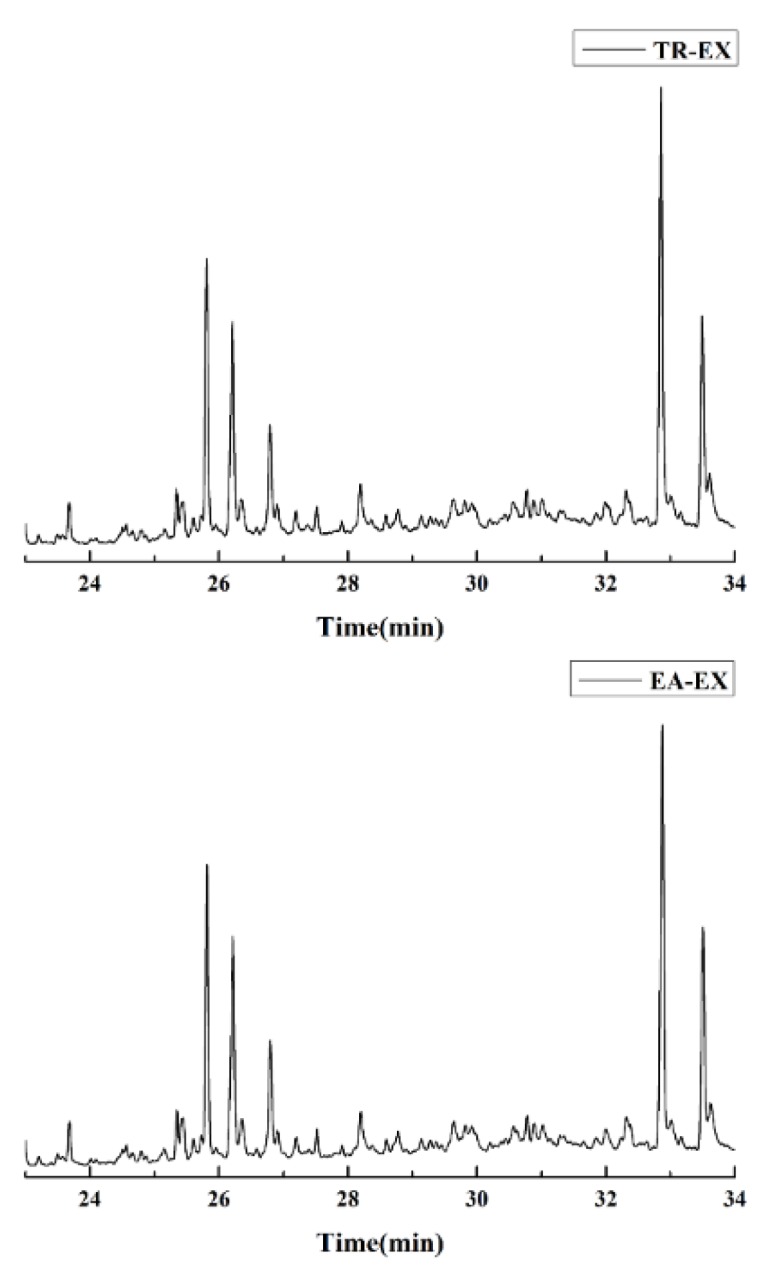
Gas chromatography-mass spectrometry (GC-MS) chromatograms of ethyl acetate extracts (EA-EX) and trichloromethane extracts (TR-EX) from *Phoebe zhennan* wood residues.

The yield of the ethyl acetate extracts (EA-EX) and trichloromethane extracts (TR-EX) from the *Phoebe zhennan* wood residues was 3.80 ± 0.29% and 2.75 ± 0.80%, respectively. The chemical constituents of the EA-EX and TR-EX from *Phoebe zhennan* wood were analyzed by GC-MS, and the identified compounds are shown in Table 1. Ten common compounds were detected in the two extracts, accounting for about 45% of all the detected substances. The GC-MS chromatograms and the major compounds of the two extracts are presented in Figure 1. The relative percentages of b-Guaiene, 2-isopropyl-5-methyl-9-methylene[4.4.0]dec-1-ene,2,3,3,4,7-pentamethyl-2, 3-dihydro-benzofuran, 3,7-benzofurandiol,2,3-dihydro-2,2-dimethyl- and N-alpha-BOC-L-lysine in EA-EX were 11%, 13%, 6%, 19% and 10%, respectively. The relative percentages of eremophilene (7CI), 2-isopropyl-5-methyl-9-methylene[4.4.0]dec-1-ene, cycloocta-1,3,6-triene, 2,3,5,5,8,8-hexamethyl-, 3,7-benzofurandiol,2,3-dihydro-2,2-dimethyl- and 2-methyl-5-(2,6,6-trimethyl-cyclohex-1-enyl)-pentane-2,3-diol in TR-EX were 11%, 13%, 6%, 20% and 10%, respectively. 

**Figure 2:**
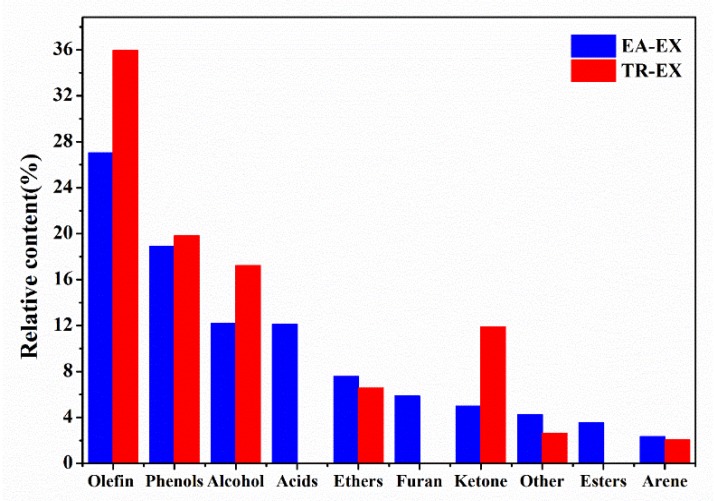
The relative content of compound species in ethyl acetate extracts (EA-EX) and trichloromethane extracts (TR-EX) from *Phoebe zhennan* wood.

In the two extracts, olefins, phenols and alcohols were the main substances (Figure 2). For comparison, the content of olefins was relatively high, and the content of aromatic hydrocarbons was the lowest. The content of olefins, phenols, alcohol, and ketone in the TR-EX was higher than in the EA-EX; however, acids and esters were found in the EA-EX, which were not found in the TR-EX. This may be due to the fact that ethyl acetate can dissolve acids and esters.

### 2.2. FTIR

**Figure 3:**
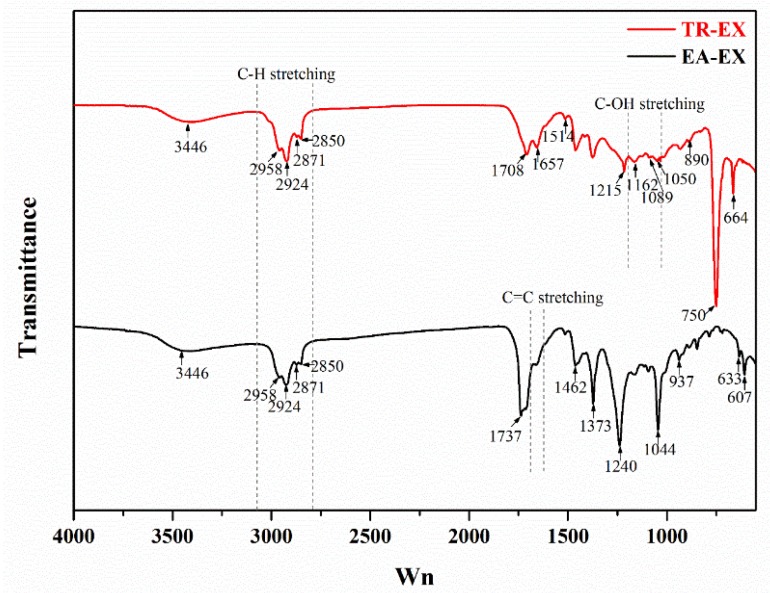
Fourier transform infrared spectroscopy (FTIR) spectra of ethyl acetate extracts (EA-EX) and trichloromethane extracts (TR-EX) from *Phoebe zhennan* wood.

The FTIR spectra of the EA-EX and TR-EX from *Phoebe zhennan* wood are illustrated in Figure 3. The bands at around 3446 and 2924 cm^−1^ attributed to O-H stretching vibration and C-H stretching vibration, respectively, were both found in the spectra of EA-EX and TR-EX [34]. In the FTIR spectrum of EX-EX, the peak appeared at 1737 cm^−1^ was attributed to the stretching vibration of carbonyl. The peak at 1666 cm^−1^ represented the C=C stretching vibration of olefins. The peak at 1462 cm^−1^ and 1373 cm^−1^ was ascribed to asymmetric angle and symmetrical angle of CH_3_, respectively. The peak at 1240 cm^−1^ represented C-O-C stretching vibration of aromatic ether. The strong absorption peak appearing at 1044 cm^−1^ was due to C-O-C symmetrical stretching vibration of fatty acid ester, while there was no such absorption on the spectrum of the TR-EX because of the absence of the acid ester substances as identified by GC-MS. The peak appearing at 937 cm^−1^ represented the outward bending vibration of the carboxylic acid C-OH. On the FTIR spectrum of the TR-EX, the peak appearing at 1708 and 1657 cm^−1^ represented C=O stretching vibrations of ketone carbonyl and quinone carbonyl, respectively. The peak at 1514 cm^−1^ was due to C=C stretching vibration of the aromatic nucleus. The peak appearing at 1215 cm^−1^ represented C-OH stretching vibration of phenols. The peak at 1162 cm^−1^ was ascribed to the C-OH stretching vibration of alcohol. The peak appearing at 890 cm^−1^ was attributed to the CH_2_ wagging vibration of olefin. The strong absorption peak at 750 cm^−1^ represented the out-of-plane C=CH bending vibration of the benzene ring. The peak at 664 cm^−1^ was ascribed to the out-of-plane bending vibration of alcohol. The FTIR spectra of the two extracts further gave evidence of the substances identified by GC-MS. 

### 2.3. Thermal Behavior

**Figure 4:**
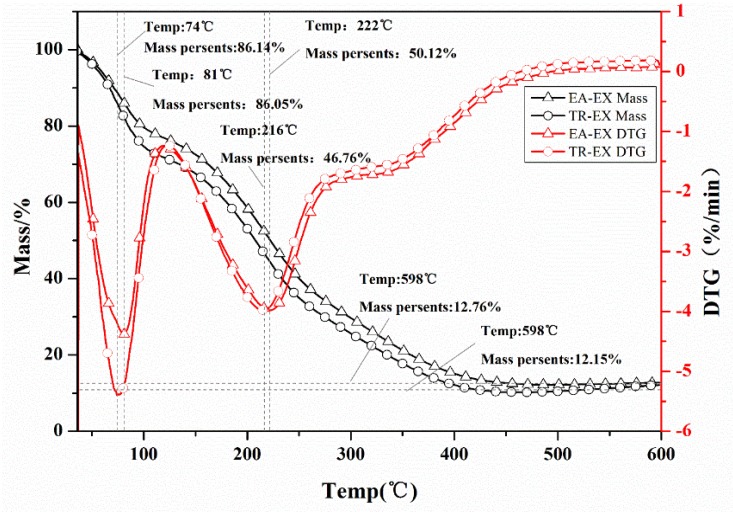
Thermogravimetric (TG) and differential thermogravimetric (DTG) curves of ethyl acetate extracts (EA-EX) and trichloromethane extracts (TR-EX) from *Phoebe zhennan* wood.

The TG and differential thermogravimetric (DTG) analysis of EA-EX and TR-EX from *Phoebe zhennan* wood are presented in Figure 4. The TG curve showed that the thermal degradation of EA-EX included two stages. In the first stage, there was an initial weight loss (about 15% weight loss) resulting in a peak temperature at 81 °C as indicated by the DTG curve, mainly due to the evaporation of moisture and a small amount of volatile substances in the EA-EX. In the second stage, the mass was significantly degraded and the mass percentage dramatically decreased to 12.76%. The DTG curve indicated that the maximum rate of weight loss occurred at 222 °C, mainly because the pyrolysis temperature of the main components such as 4,6,6-trimethyl-bicyclo [3.1.1] hept-3-en-2-one, 1-Methylbicyclo [3.2.1] octane are around 200–230 °C, and the degradation of such compounds at the mentioned temperature contributed to the dramatic weight loss. The pyrolysis process of the TR-EX was similar to that of the EA-EX despite the difference in weight loss rate and the peak temperature of the maximum weight loss rate. The peak temperatures for the TR-EX in the first and second stages were 74 °C and 216 °C, respectively, which was a little lower compared to the TR-EX. This result revealed that the thermal stability of the EA-EX was a little higher compared with that of the TR-EX; this may be due to the differences in the chemical constituents between the two extracts as stated before. 

### 2.4. UV-Vis Spectra

**Figure 5:**
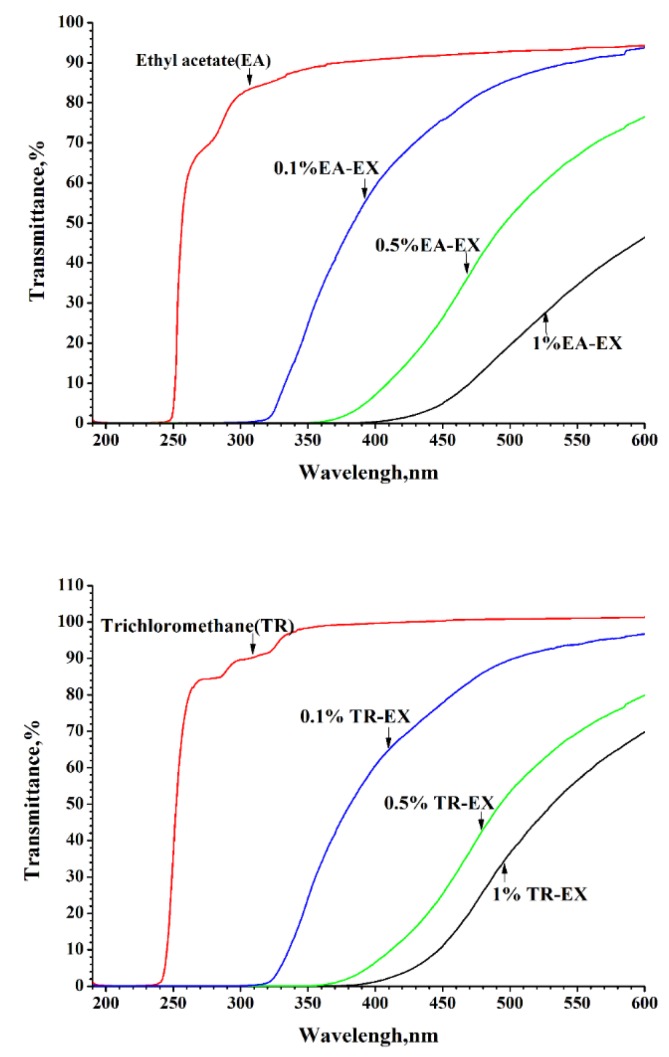
Transmittance spectra of solvents and the solvent diluted extract solutions.

Figure 5 shows the transmission spectra of ethyl acetate (EA), trichloromethane (TR) and the diluted extract solution. Ethyl acetate almost completely transmitted the UV light in the UV-B (280–315 nm) and UV-A (315–400 nm) range. However, the diluted EA-EX solution exhibited excellent UV absorption capacity, i.e., almost all of the UV-B (280–315 nm) and 70% of the UV-A (315–400 nm) radiation was absorbed by the 0.1 wt% EA-EX solution. The EA-EX extract solution blocked 100% of UV-B (280–315 nm) and 98% of UV-A (315–400 nm) when its concentration was 0.5 wt%. Almost all the UV light was blocked when the concentration of the EA-EX solution increased to 1 wt%. Similarly, the TR-EX solution also exhibited strong UV light absorption, which increased as the weight concentration increased. The TR-EX solution can also absorb all of the UV light at a concentration of 1%.

By comparing the UV light absorption between the TR-EX and EA-EX solutions, no significant difference was observed. The UV-vis spectra, as well as the chemical composition of EA-EX and TR-EX, revealed that the extracts from *Phoebe zhennan* wood had super UV shielding properties and may process great potential on the development of biobased UV shielding materials for the protection of UV-sensitive products if the extracts had considerable compatibility with the matrix materials. The UV light absorption of TR-EX and EA-EX solutions may be due to their chemical compositions, as identified in GC-MS. Commonly, the aromatic ring as well as C=C, C=O and -C=C-C=C- have π bond electrons. After absorbing energy, a π–π* transition occurs, and the maximum absorption peak wavelength of the compound enters the near-ultraviolet region. Therefore, the UV absorption of the extracts from *Phoebe zhennan* wood may be due to the aromatic rings in the compounds such as 2,3-dihydro-2,2-dimethyl-3,7-benzofurandiol; 5-allyl-6-(allyloxy)-1,3-benzodioxole and 2,3,3,4,7-pentamethyl-2,3-dihydro-benzofuran. Previous research revealed that the benzene ring structure in the diethyl ether extracts was mainly from the identified compound of 3,7-benzofurandiol,2,3-dihydro-2,2-dimethyl [33]; this result indicated that the number in compounds providing benzene ring structure in EA-EX and TR-EX was larger, which guaranteed the UV light absorption of the EA-EX and TR-EX.

### 2.5. Preparation and Characterization of the UV-Shielding Films

**Figure 6:**
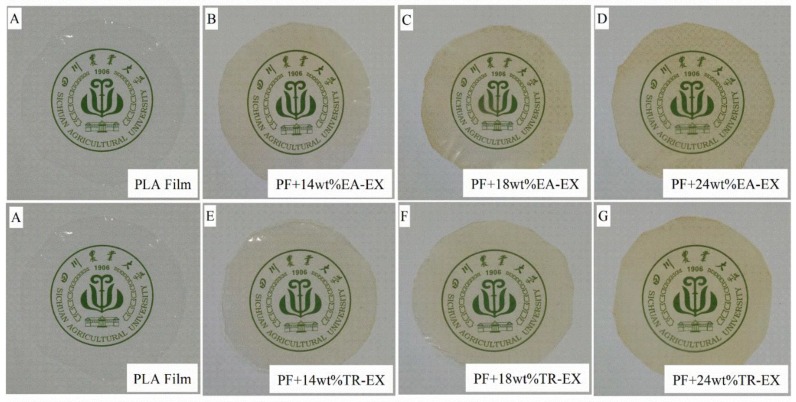
Photograph images of the PLA film (PF): (**A**), UV shielding films (UV-PF) with 14 wt% EA-EX (**B**), UV-PF with 18 wt% EA-EX (**C**), UV-PF with 24 wt% EA-EX (**D**), UV-PF with 14 wt% TR-EX (**E**), UV-PF with 18 wt% TR-EX (**F**) and UV-PF with 24 wt% TR-EX (**G**).

Based on the super UV absorption and the thermal stability of the extracts, they were used as UV absorbers and added into the PLA matrix in order to fabricate films with UV shielding property. The photographs of the fabricated neat PLA film (PF) and the two types of UV shielding films (UV-SF) with different extractive content are shown in Figure 6. The UV-SF prepared from either EA-EX or TR-EX was highly transparent. The visible difference between the PF and the UV-SF was that the UV-SF exhibited a slightly yellow color, and when increasing the extracted content, the color slightly became darker. However, no heterogeneous substances were observed on the surface of the films, indicating that the extracts were well mixed with the matrix and had good compatibility with PLA.

**Figure 7:**
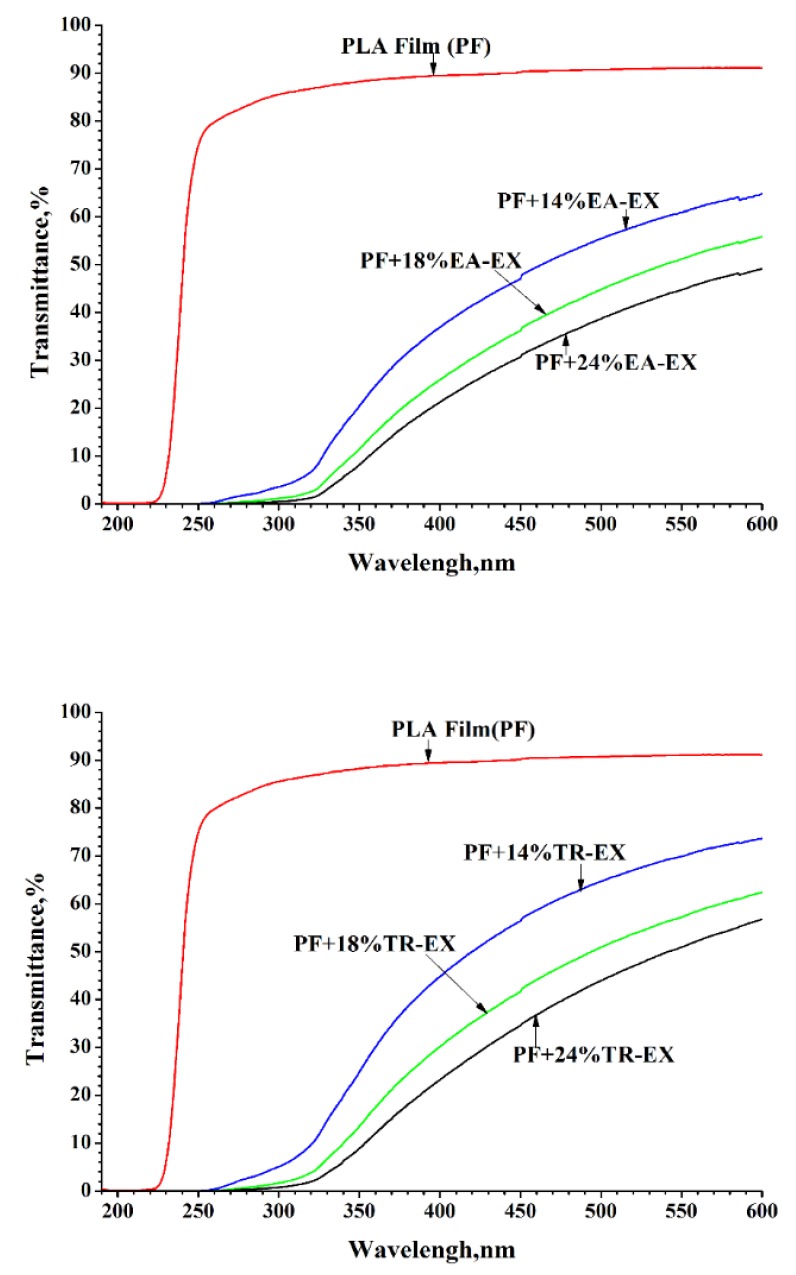
Transmittance spectra of the neat PLA film and the UV-shielding films.

As shown in Figure 7, the PF showed no transmission of UV light in the lower range of UV-C (190–230 nm), and almost transmitted the UV light in the higher range of UV-B (280–315 nm) and UV-A (315–400 nm). After the addition of EA-EX into PLA, the UV-PF exhibited strong UV blocking ability and 96% of UV-B (280–315nm) and 80% of UV-A (315–400 nm) was blocked by UV-PF with 14 wt% of EA-EX. The film blocked almost all of UV-B (280–315 nm), 87% of UV-A (315–400 nm) when the content of the extract was 18%. The addition of 24 wt% extract into UV-PF resulted in the blocking of 100% of UV-B (280–315 nm) and 89% of UV-A (315–400 nm). Figure 7 also shows that the addition of TR-EX could also contribute to the UV blocking ability of PLA, and the UV blocking ability of the UV-PF increased as the extracted content increased. From the transmission spectra of PF and UV-PF, it can be seen that the addition of the EA-EX and TR-EX from *Phoebe zhennan* wood into PLA can significantly improve the UV blocking ability of the PLA film and the UV-PF could almost completely block UV-B. For comparison, the addition of EA-EX resulted in higher absorption ability in UV-A than that of TR-EX. The absorption of UV-A light for UV-PF with the addition of 14% of EA-EX and TR-EX was 80% and 74%, respectively. The addition of 14% of diethyl ether extracts into a polylactic acid matrix could block 75% of UV-A, which has similar absorption ability as that of TR-EX [33]. This result revealed that EA-EX was more prefered in the preparation of UV shielding films.

**Figure 8:**
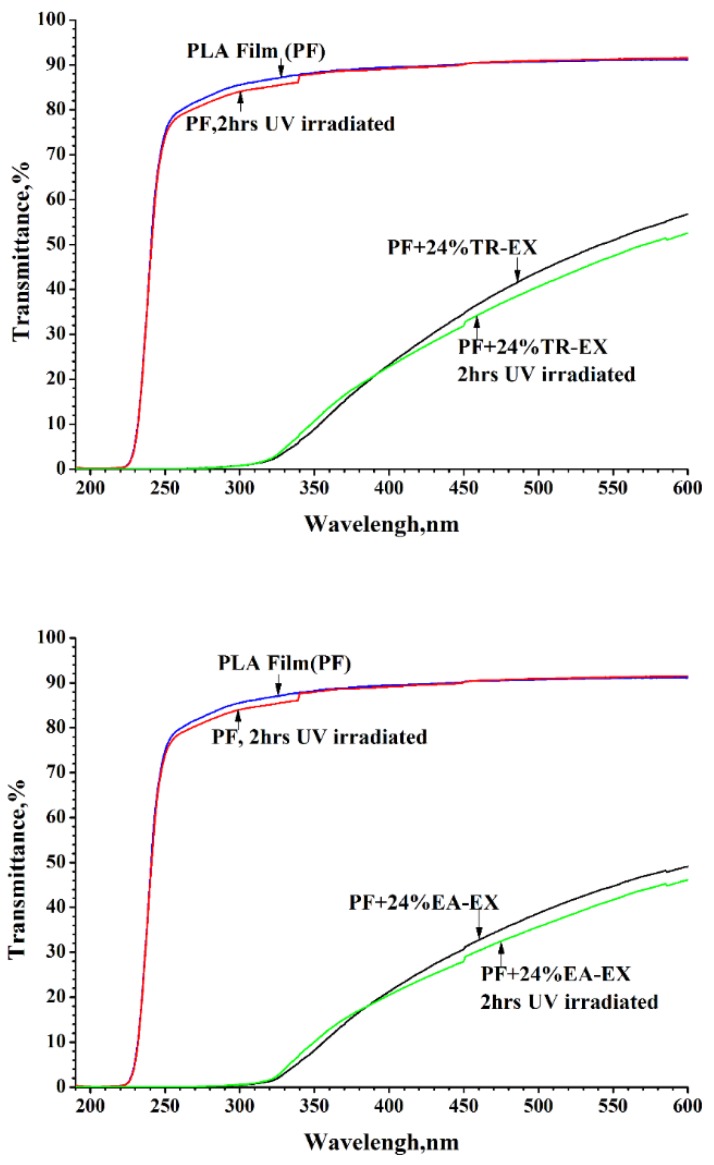
Comparison in the UV stability of the neat PLA film (PF) and the UV shielding film (UV-SF) with 24% extractive under strong UV irradiation.

An important factor affecting the performance and service life of biobased UV shielding materials is the stability of UV-PF under UV light irradiation. The PF and UV-PF were irradiated under a UV xenon lamp, and the UV absorption was measured. After UV lamp irradiation, the light transmittance of the PF slightly decreased in the range of 250-350 nm (Figure 8). The transmittance of UV-SF containing 24 wt% of extract (PF + 24% EA-EX and PF + 24% TR-EX) did not change in the UV-B range, indicating that the film was stable in the UV-B range. The absorption of the UV-A light for the PF with 24% EA-EX and PF with 24% TR-EX was only reduced by 1%. Meanwhile, the light transmittance in the visible light region was also slightly reduced after UV lamp irradiation. Compared to cellulose-lignin biodegradable UV protection film as previously reported [35], after the lamp irradiation, the reduction in UV absorption of the UV-PF prepared in this study was smaller. This result indicated that the strong UV lamp had minimal influence on the UV stability of UV-SF.

**Figure 9:**
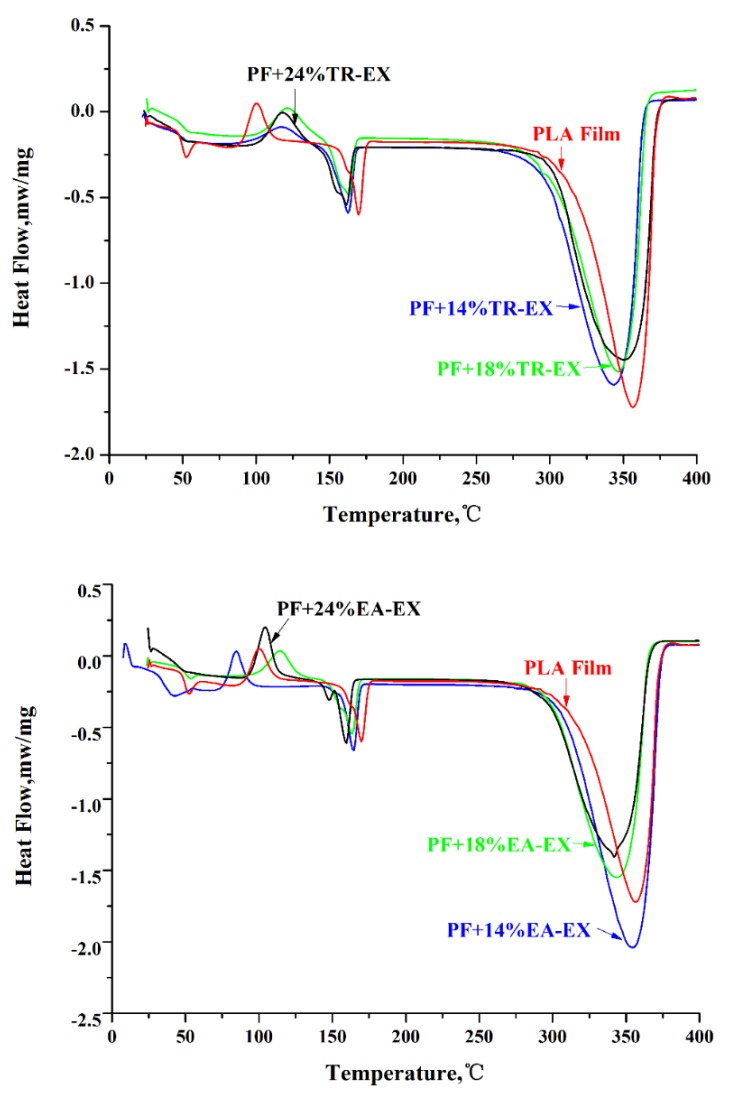
DSC of the PF and the UV-SF with different extracts content.

The differential scanning calorimetry (DSC) curves for PF and UV-SF with different extract contents are shown in Figure 9, and Table 2 shows the DSC data. The melting temperature (T_m_) of PF was about 169 °C, and the T_m_ of UV-SF was lower than the T_m_ of PF. The temperature of the maximum thermal degradation rate (T_d_) of PF was about 356 °C, and the temperature of PF was higher than the T_d_ of UV-SF containing different extract contents. Although the degradation temperature of PF was higher than the degradation temperature of UV-SF, the difference was not significant and will not affect the end use of the product. The extracts had a lower degradation temperature than PLA because of the low molecular weight component as detected by GC-MS, which contributed to the relatively low thermal stability of the UV-SF. 

The tensile strength and elongation at the break of PF and the different UV-SF are listed in Table 3. The tensile strength of the PLA film was 30.73 ± 3.00 MPa, and the elongation at break was 11.87 ± 3.36%. The tensile strength and elongation at break were increased when a small amount of EA-EX was added to the PLA matrix. When the relative content of the EA-EX reached a certain level, the tensile strength and elongation at break were reduced. The addition of TR-EX also reduced the tensile strength. For comparison, the decrement in tensile strength induced by TR-EX was larger than that by EA-EX. The difference in the decrement in tensile strength induced by the two extractives may be due to the differences in the chemical constituents and the properties of each compound. 

## 3. Materials and Methods

### 3.1. Materials and Chemicals

**Figure 10:**
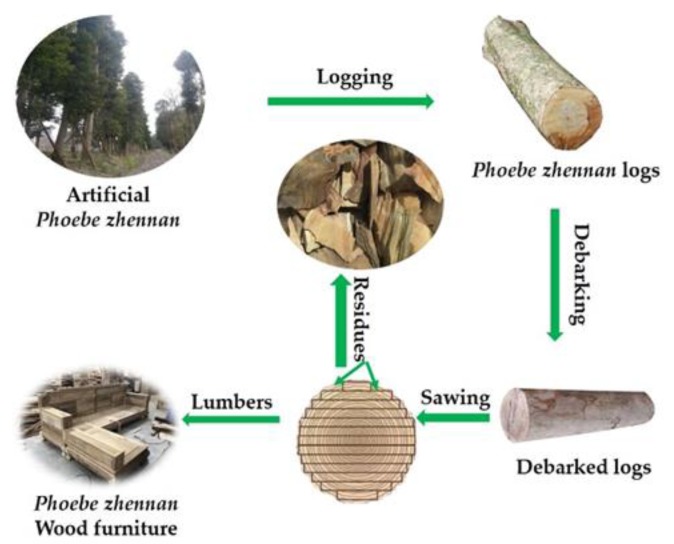
Flowchart of the saw milling process and the residue sample portions in the *Phoebe zhennan* wood.

*Phoebe zhennan* wood processing residues were obtained from sawmilling in a local furniture factory located in Ya’an, China. The lumber sawing process and the portions of the collected samples is illuminated in Figure 10. The residue samples were mainly produced from the sapwood of the *Phoebe zhennan* wood logs. The samples were air-dried and reduced to particles using a laboratory mill. The particles with size of 40–60 mesh were collected and then dried to a constant weight in an oven maintained at 60 °C. The dried particles were stored in sealed bags and used without further treatment. The density and melt flow index of the PLA matrix were 1.24 g/cm^3^ and 14 g/10 min at 210 °C /2.16 kg, respectively. All chemicals used in this study were of analytical grade and obtained from commercial sources.

### 3.2. Preparation of the Extracts

The ethyl acetate extracts (EA-EX) and trichloromethane extracts (TA-EX) of *Phoebe zhennan* wood residues were prepared by using the Soxhlet extraction method. Twenty grams of the residue samples were extracted with 225 mL ethyl acetate and trichloromethane, respectively. The extraction was maintained for 6 h until the color of the solvent became colorless and transparent, thereafter, the extract in the flask was vacuum-filtered to remove particulate impurities. Then, the filtration was condensed at 65 °C using a rotary evaporator equipped with a vacuum to remove ethyl acetate and trichloromethane, respectively. The condensed extracts after evaporation were cream-like and were stored in the refrigerator(4 °C) for further use.

### 3.3. Preparation of the UV Shielding Films (UV-SF)

A simple solvent cast method was used to prepare the UV-SF. The main procedure for the preparation of the films was as follows: 0.2 g of PLA and 20 mL trichloromethane was added in a 50 mL beaker. The mixture was maintained under stirring with a magnetic stirrer. After the complete dissolution of the PLA, a designated amount of the ethyl acetate extracts or the trichloromethane extracts was added to the mixture followed by slowly stirring for 15 min, thereafter, the solution in the beaker was poured into a glass petri dish with a diameter of 10 cm. The thickness of all the films was controlled by adding the same dry weight of the mixture in the petri dish. After the evaporation of the solvent, the films were peeled off the petri dish and cured at room temperature for 24 h prior to characterization. The neat PLA film (PF) was prepared with the same procedure without the addition of any extracts. A total of seven groups of the PF and UV-SF with different percentages of extracts and fifteen samples for each group were fabricated.

### 3.4. UV-Vis Spectra 

The optical properties of the *Phoebe zhennan* wood residues extract, the PF and the UV-SF were measured using a UV-Vis spectrophotometer (UNICO UV-4802H, Shanghai, China). Both EA-EX and TR-EX were diluted to a concentration gradient of 0.1%, 0.5% and 1.0% with ethyl acetate and trichloromethane, respectively. Prior to the test, distilled water was used to calibrate the baseline. Ethyl acetate and trichloromethane were used as blank control. The spectra were collected in the scanning range 190–800 nm at the scanning speed of 1.0 nm. The PF and UV-SF samples were cut into pieces with the same size as the cuvette. The dry blank cuvette was used to calibrate the baseline. The neat PF film sample was used as blank control. The determination process was the same as that for the extracts solutions. 

### 3.5. UV Irradiation of the Films

The UV light stability of the UV shielding films under UV lamp irradiation was evaluated in accordance with a referenced method [35]. The neat PLA films and the UV-shielding film samples were irradiated under strong UV light for 2 h by using a 200 W Xe–Hg lamp. The distance from the sample to the UV lamp was 25 cm. After the UV lamp irradiation, the UV absorbance properties of the irradiated samples were measured. 

### 3.6. Gas Chromatograph Mass Spectrometer (GC-MS)

The identification of the chemical components of the ethyl acetate extracts (EA-EX) and trichloromethane extracts (TA-EX) of *Phoebe zhennan* wood residues were performed by using a gas chromatograph (GC; Agilent 7890A, Shanghai, China) and a mass spectrometry (MS; Agilent 5975C VL MSD, Shanghai, China) equipped with a fused capillary column (HP-5) with 95% dimethylpolysiloxane and 5% phenyl as the stationary phase. The injection mode was split at a split rate of 35, the column was kept at 30 °C for 2 min and then at a rate of 10 °C/min it was heated to 250 °C and maintained for 10 min. The carrier gas was helium at a flow rate of 1.5 mL/min [36].

### 3.7. Fourier Transform Infrared Spectroscopy (FTIR)

The FTIR analysis was performed using a Nicolet Nexus 670 spectrometer (Dongguan, China) equipped with a thermo nicolet Golden Gate MKII single reflection ATR accessory (Dongguan, China). A small amount of condensed extracts after evaporation was applied directly to the diamond crystal. Data collection was performed with a 4 cm^−1^ spectral resolution, and 32 scans were taken per sample.

### 3.8. Thermogravimetric (TG)

Thermogravimetric (TG) and differential thermogravimetric (DTG) analysis were conducted with a thermal analyzer, TGA (Q50) (TA Instruments, New Castle, DE, America), to simultaneously obtain thermogravimetric data. About 2 mg of condensed extracts after evaporation was analyzed by the thermal analyzer. Pyrolysis was terminated at 600 °C with a heating rate of 20 °C /min under a flow of 60 mL/min of nitrogen gas [37].

### 3.9. Differential Scanning Calorimetry (DSC) 

The thermal stability of the neat PLA and UV protective films was analyzed with a differential scanning calorimetry thermal analyzer (DSC; Q250, Shanghai, China). About 5 mg of the samples were used. The samples were preheated to 200 °C, then cooled down to 25 °C. The heating was terminated at 450 °C with a heating rate of 20 °C /min under a 40 mL/min flow of nitrogen gas.

### 3.10. Mechanical Property

The test of the tensile strength and elongation at break of the PLA and the UV protective films was performed using a tensile strength test machine (XLW-PC, Jinan, China) according to Chinese standard method of GB1040.3 [38]. The film samples with the dimension of specimen used for the experiments were sample strips of 50 mm × 5mm × 0.03 mm were prepared. The load and the cross speed for the test were 5 KN and 2 mm/min. Three replicates for each group of films were tested.

## 4. Conclusions

The chemical compositions of the ethyl acetate and trichloromethane extracts from *Phoebe zhennan* wood were identified by GC-MS, and olefins, phenols and alcohols were the main substances of the extracts. The FTIR spectra further confirmed the chemical constituents of the extracts. The diluted extracts solutions in both solvents showed UV blocking properties, and the extract solution with 1 wt% could almost block all the UV-C (230–280 nm), UV-B (280–315 nm), and UV-A (315–400 nm) light. UV-shielding films (UV-SF) were successfully prepared by adding the two extracts into PLA. The UV-SF with the addition of 24 wt% of the extracts could block 100% of the UV-B (280–315 nm) and 89% of the UV-A (315–400 nm). After strong UV irradiation, the fabricated UV-SF still exhibited super UV shielding performance indicating the UV-SF had good UV stability. Though the addition of extracts could somewhat decrease the thermal stability, the decrease was insignificant and its effect on the end-use of the film can be ignored. The tensile test results showed the addition of the extracts would decrease the mechanical strength of the UV-SF, and the EA-EX had less effect on tensile properties than TR-EX as the extracts conttent reached 18%. The results of this research show that ethyl acetate and trichloromethane extracts from *Phoebe zhennan* wood could be used as UV absorbers for the preparation of UV shielding films.

## Figures and Tables

**Table 1:** Chemical compositions of *Phoebe zhennan* wood residue extracts

S/N	Compound	Formula	Relative Content (%)		Structural Formula
			Ethyl Acetate	Trichloro-Methane	
1	4,6,6-Trimethyl-bicyclo[3.1.1]hept-3-en-2-one	C_10_H_14_O	0.81	0.73	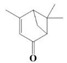
2	β-Cubebene	C_15_H_24_	1.1	1.14	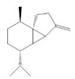
3	l-Calamenene	C_15_H_22_	1.19	0.98	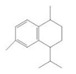
4	a-Elemol	C_15_H_26_O	1.69	1.76	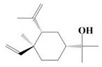
5	Naphthalene,1,2,3,4,4a,7-hexahydro-1,6-dimethyl-4-(1-methylethyl)-	C_15_H_24_	1.14	1.08	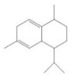
6	2-Isopropyl-5-methyl-9-methylene[4.4.0]dec-1-ene	C_15_H_24_	13.24	13.11	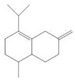
7	3-Isolongifolol	C_15_H_26_O	1.49	1.07	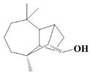
8	2,3-dihydro-2,2-dimethyl-3,7-benzofurandiol	C_10_H_12_O_3_	18.91	19.82	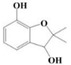
9	beta-Eudesmol	C_15_H_26_O	1.65	1.67	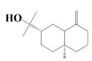
10	1,4-Methanoazulen-7(1H)-one, octahydro-4,8,8,9-tetramethyl-, (+)-	C_15_H_24_O	3.2	4.08	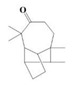
11	Elixene	C_15_H_24_	0.46	_	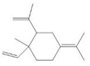
12	Agarospirol	C_15_H_26_O	1.88	_	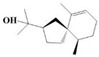
13	(+)-g-Eudesmol	C_15_H_26_O	2.47	_	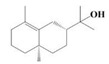
14	α-Cuparenol	C_15_H_22_O	0.94	_	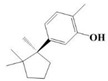
15	b-Guaiene	C_15_H_24_	11.25	_	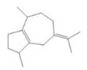
16	2,3,3,4,7-Pentamethyl-2,3-dihydro-benzofuran	C_13_H_18_O	5.87	_	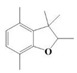
17	1-Methylbicyclo[3.2.1]octane	C_9_H_16_	1.71	_	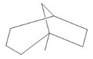
18	Culmorin	C_15_H_26_O_2_	1.16	_	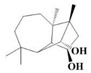
19	Ethylphosphonic acid methylethyl ester	C_5_H_12_O_3_P	1.11	_	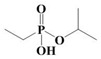
20	cis-Z-α-Bisabolene epoxide	C_15_H_24_O	2.57	_	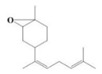
21	Aromadendrene oxide-(2)	C_15_H_24_O	1.55	_	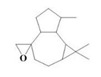
22	(-)-delta.-Panasinsine	C_15_H_24_	0.98	_	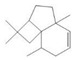
23	2-Heptyne, 7-bromo-	C_7_H_11_Br	2.51	_	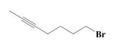
24	Diepicedrene-1-oxide	C_15_H_24_O	1.83	_	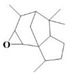
25	5-Methyl-2-ethenyl-cyclohexane-1-carboxylic acid	C_10_H_16_O_2_	2.46	_	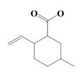
26	Norketone	C_14_H_20_O_3_	1.2	_	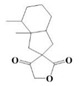
27	N,N-Dimethyl methylphosphoramidofluoridate	C_3_H_9_FNOP	0.99	_	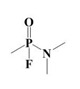
28	2,2,6,7-Tetramethyl-10-oxatricyclo[4.3.1.0(1,6)]decan-5-ol	C_13_H_22_O_2_	0.91	_	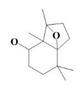
29	2-(1,4,4-Trimethylcyclohex-2-en-1-yl)ethyl p-toluenesulfonate	C_18_H_26_O_3_S	1.46	_	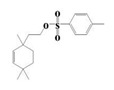
30	5-Allyl-6-(allyloxy)-1,3-benzodioxole	C_13_H_14_O_3_	1.62	_	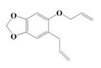
31	2(1H)Naphthalenone,3,5,6,7,8,8a-hexahydro-4,8a-dimethyl-6-(1-methylethenyl)-	C_15_H_22_O	0.97	_	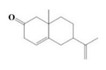
32	N-alpha-BOC-L-Lysine	C_11_H_22_N_2_O_4_	9.66	_	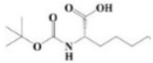
33	g-Selinene	C_15_H_24_	_	1.85	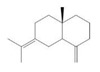
34	γ-Himachalene	C_15_H_24_	_	2.16	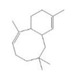
35	d-Longifolene	C_15_H_24_	_	0.85	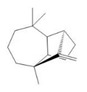
36	Eremophilene (7CI)	C_15_H_24_	_	11.27	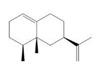
37	Cycloocta-1,3,6-triene,2,3,5,5,8,8-hexamethyl-	C_14_H_22_	_	5.57	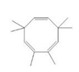
38	Spiro[2.5]octane,5,5-dimethyl-4-(3-oxobutyl)-	C_14_H_24_O	_	1.78	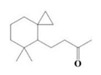
39	Dihydrocarveol	C_10_H_18_O	_	0.93	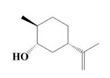
40	cis-(Z)-α-Bisabolene epoxide	C_15_H_24_O	_	5.12	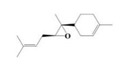
41	Alloaromadendrene oxide-(1)	C_15_H_24_O	_	2.17	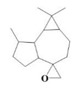
42	3,7,11-Trimethyl-dodeca-2,4,6,10-tetraenal	C_15_H_22_O	_	2.64	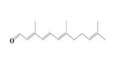
43	3,4,4-Trimethyl-3-(3-oxo-but-1-enyl)-bicyclo[4.1.0]heptan-2-one	C_14_H_20_O	_	1.65	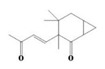
44	Cyclopentanecarboxaldehyde,2-methyl-3-methylene-	C_8_H_12_O	_	1.61	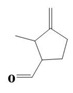
45	Isoaromadendrene epoxide	C_15_H_24_O	_	0.87	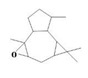
46	diepi-α-Cedrene epoxide	C_15_H_24_O	_	0.94	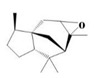
47	Isopulegol	C_10_H_18_O	_	1.73	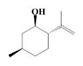
48	2-Methyl-5-(2,6,6-trimethyl-cyclohex-1-enyl)-pentane-2,3-diol	C_15_H_28_O_2_	_	10.07	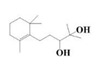

**Table 2:** DSC results for PF and the UV-SF with extracts.

Films	T_m_ (°C)	T_d_ (°C)
Neat PLA film (PF)	169	356
PF + 14% EA-EX	164	354
PF + 18% EA-EX	163	343
PF + 24% EA-EX	159	341
PF + 14% TR-EX	162	343
PF + 18% TR-EX	162	346
PF + 24% TR-EX	161	350

**Table 3:** Tensile strength and elongation at break of the PF and the UV-SF with different extracts content.

Films	Tensile Strength (MPa)	Elongation at Break (%)
Neat PLA film (PF)	30.73 ± 3.00	11.87 ± 3.36
PF + 14% EA-EX	32.49 ± 2.45	13.89 ± 2.48
PF + 18% EA-EX	30.73 ± 3.26	12.62 ± 1.31
PF + 24% EA-EX	28.39 ± 1.02	10.60 ± 0.87
PF + 14% TR-EX	28.60 ± 1.72	13.94 ± 0.10
PF + 18% TR-EX	28.28 ± 3.24	13.80 ± 1.59
PF + 24% TR-EX	25.34 ± 1.08	13.35 ± 2.51
